# Optimization of Paper-Based Alveolar-Mimicking SERS Sensor for High-Sensitivity Detection of Antifungal Agent

**DOI:** 10.3390/bios14120566

**Published:** 2024-11-22

**Authors:** Hyunjun Park, Kyunghwan Chai, Eugene Park, Woochang Kim, Gayoung Kim, Joohyung Park, Wonseok Lee, Jinsung Park

**Affiliations:** 1 Department of Biomechatronic Engineering, College of Biotechnology and Bioengineering, Sungkyunkwan University, Suwon 16419, Republic of Korea; guswns1105@g.skku.edu (H.P.); delorean12@g.skku.edu (E.P.); wkim91@skku.edu (W.K.); 2 Department of Biopharmaceutical Convergence, Sungkyunkwan University, Suwon 16419, Republic of Korea; kyungh2012@g.skku.edu (K.C.); kgy9173@g.skku.edu (G.K.); 3 Department of Electrical Engineering, Korea National University of Transportation, Chungju 27469, Republic of Korea; 4 Department of MetaBioHealth, Sungkyunkwan University, Suwon 16419, Republic of Korea

**Keywords:** crystal violet, bioinspired, paper-based, Raman spectroscopy, surface-enhanced Raman scattering (SERS), high sensitivity

## Abstract

Crystal violet (CV) is a disinfectant and antifungal agent used in aquaculture that plays a vital role in treating aquatic diseases and sterilizing water. However, its potential for strong toxicity, including carcinogenicity and mutagenicity, upon accumulation in the body raises concerns regarding its safe use. Therefore, there is a growing need for the quantitative detection of CV in its early application stages to ensure human safety. Recently, Raman spectroscopy-based surface-enhanced Raman scattering (SERS) detection research has been actively conducted; consequently, an alveolar-mimicking SERS paper (AMSP) inspired by the structure of the human lungs was developed. The AMSP was optimized through various factors, including paper type, reducing agent, reducing agent concentration, and reaction time. This optimization enhanced the surface area of interaction with the target substances and promoted hotspot formation, resulting in enhanced SERS performance. The substrate exhibited exceptional uniformity, reproducibility, and reliability. CV was successfully detected at a concentration of 1 nM in laboratory settings. Furthermore, the AMSP detected CV at 1 nM in real-world environmental samples, including fish farm water and human serum, confirming its potential as a practical detection and monitoring platform for CV in real-world samples.

## 1. Introduction

Crystal violet (CV), a dye belonging to the triphenylmethane group, is used in various of chemical, biological, and industrial applications. In the medical field, CV helps control fungal infections and intestinal parasites and serves as an antifungal agent for burn victims [1]. Industrially, CV is employed as an antimicrobial solution to prevent mold growth in poultry feed and as a disinfectant and antifungal agent in aquaculture, where it is used to treat diseases in aquatic animals and sterilize water [2,3]. However, despite its widespread use, CV is highly toxic and has been associated with carcinogenic and mutagenic effects in humans, raising concerns regarding its use in aquaculture [4,5]. Specifically, when exposed to large amounts of CV in the body, it has been shown to accumulate in muscles, kidneys, liver, and other tissues, producing leucocrystal violet (LCV) as a metabolic byproduct [6,7]. Therefore, the quantitative detection of CV in its early application stages is crucial to ensure human safety in both environmental and medical contexts.

Consequently, research is actively focused on developing monitoring platforms to detect trace levels of CV in environmental samples. Over the past few decades, numerous platforms have been developed to address these issues. High-performance liquid chromatography (HPLC) [8,9], liquid chromatography coupled with mass spectrometry (LC-MS) [10,11], UV–visible spectroscopy (UV-vis) [12,13], electrochemical sensors [14,15,16,17], and fluorescence analysis [18,19] have been utilized widely in CV detection studies. Among these techniques, LC-MS is the most commonly adopted. However, LC-MS is limited by its complex sample preparation procedures and high sample consumption. Recently, the Raman spectroscopy-based surface-enhanced Raman scattering (SERS) detection platform has attracted considerable attention in the fields of environmental pollutant detection and medical diagnosis due to its exceptional sensitivity and straightforward sampling techniques [20,21,22,23]. However, current SERS-based platforms face limitations due to the complex design and lengthy fabrication time required to achieve the uniform arrangements of metal nanostructures essential for the SERS effect [24,25,26,27]. To address these challenges, it is essential to develop substrates with numerous nanogaps to maximize the SERS effect while simplifying the fabrication design.

In this study, human lungs and alveoli were used as inspiration to achieve efficient SERS performance. The human lung has a branching structure that divides into bronchi, maximizing the pathways through which air can reach the alveoli, thereby enhancing the efficiency of gas exchange [28,29]. Additionally, the lungs comprise millions of small air sacs called alveoli, dramatically increasing the surface area and, consequently, the efficiency of gas exchange within the body [30,31]. Inspired by this structure, an alveolar-mimicking SERS paper (AMSP) with a simple design was fabricated using cellulose acetate (CA) paper, in which gold nanoparticles were grown and functionalized; this approach was optimized to maximize the interaction area with the target substances and improve hotspot generation, thereby enhancing the SERS effect. The fabricated AMSP was optimized by varying several parameters, including the substrate type, the type and concentration of the reducing agent used for gold nanoparticle growth, and the reaction time. These adjustments were performed to identify the conditions that yielded the most effective SERS performance. This study confirmed the high-sensitivity detection of CV using AMSP. Furthermore, the detection capability of CV was verified in aquaculture water and human serum, where CV contamination is a concern. These findings demonstrate the potential of the AMSP as a sensitive on-site detection platform for CV and other harmful substances in the environment.

## 2. Experimental Section

### 2.1. Materials and Reagents

CA paper (Φ = 55 mm) was obtained from Hyundai Micro (Seoul, Repulic of Korea). Cellulose and glass fiber papers (Whatman filter paper) were purchased from Sigma-Aldrich (Burlington, MA, USA). O-methoxyamine hydrochloride (OMH), trisodium citrate (TSC), ascorbic acid (AA), hydroquinone (HQ), gold (III) chloride hydrate (HAuCl_4_), rhodamine 6G (R6G), 4-aminothiophenol (4-ATP), and human serum were purchased from Sigma-Aldrich. A pH 10 buffer and 99.5% anhydrous ethanol were acquired from Daejung Reagents (Siheung, Repulic of Korea). All aqueous solutions were prepared with Millipore deionized water (DI water) with a resistance of 18.2 MΩ at 25 °C. Aquaculture water was obtained from a fish farm at Sungkyunkwan University (Suwon, Repulic of Korea).

### 2.2. Fabrication of Paper-Based SERS Substrate

The method for fabricating paper-based SERS substrates was refined and modified based on a previous study [32]. A hole puncher (Φ = 6 mm) was utilized to make small pieces of filter paper (cellulose filter paper, cellulose acetate paper, and glass fiber paper). The pieces of the paper were washed with ethanol and rinsed with DI water. The pieces were then laid on a Petri dish and dried in the oven at 60 °C. A reducing agent (20 mM; OMH, TSC, AA, or HQ) was prepared in a pH 10 buffer. The pieces were immersed in 10 mL of the reducing agent solution in a Petri dish. Next, 2 mL of HAuCl_4_ solution was slowly added. After reacting with the reducing agent, the synthesized nanoparticles were deposited onto the paper substrates, and any remaining solution was carefully removed using a pipette. Subsequently, the final paper-based substrates were thoroughly dried at room temperature (25 °C) before use.

### 2.3. Optimization for Fabrication of AMSP

To select the optimal paper type, 10 μL drops of 4-ATP at a concentration of 100 μM were dropped to SERS substrates prepared with cellulose, CA, and glass fiber paper. SERS measurements were conducted following the substrates being allowed to dry completely at room temperature.

Next, Raman measurements were conducted under consistent conditions using paper-based SERS substrates fabricated with each reducing agent (OMH, AA, HQ, and TSC) to select and optimize the type of reducing agent. For this, 10 μL drops of 100 μM R6G indicator were applied to each substrate and allowed to dry completely. Then, Raman measurements were performed to compare the Raman intensities of the indicators on each substrate.

OMH solutions ranging from 5 to 100 mM were prepared in a pH 10 buffer to optimize the concentration of OMH, which was selected as the reducing agent, and the reaction time. Pieces of the CA paper were immersed in OMH solutions of various concentrations. A 2 mL portion of a 10 mM HAuCl_4_ solution was added to each sample, and the reaction was allowed to proceed for 60 min. The reaction time was optimized by reducing HAuCl_4_ for different times (30–120 min). Subsequently, 10 μL drops of 100 μM R6G were applied to the substrates and allowed to dry completely, then Raman measurements were conducted.

Two methods—drop-casting and dipping—were compared to optimize the reaction conditions for the analyte. For the drop-casting method, sample solutions were applied to the AMSP in volumes of 2, 5, and 10 μL. In the dipping method, the same substrate was immersed in 1 mL of analyte solution. The substrates were allowed to react with the analyte for 30 min, and all the substrates were dried entirely before conducting the Raman measurements.

### 2.4. SERS Measurements on AMSP

All Raman spectra were collected using a Renishaw inVia Raman microscope (inVia Reflex, Renishaw, Wotton-under-Edge, UK). A 785 nm wavelength laser was focused with a ×20 object lens (Leica DM2700 M, Germany, and Renishaw Centrus Detector, Wotton-under-Edge, UK). SERS spectra were measured using a 785 nm laser with a power of 1.01 mW after 1 s of exposure, five accumulations (optimization), and ten accumulations (performance confirmation). All spectra were acquired in the range of 497–1623 cm^−1^. Cosmic rays were removed from each spectrum, and the polynomial baseline was subtracted using WiRE 5.1 software.

Different concentrations of R6G aqueous solutions (100 pM–100 μM) were prepared to examine the performance of the SERS substrate. Each AMSP was immersed in 1 mL of the R6G solution for 30 min. SERS measurements were conducted on the dried substrates under each condition. A 785 nm laser was selected to minimize fluorescence interference, as longer wavelengths reduce background fluorescence in SERS measurements [33].

CV was detected using SERS under three conditions: DI water, aquaculture water, and 1% human serum. In the aquaculture condition, the water was filtered twice using a 0.22 µm syringe filter and stored for 12 h to eliminate unnecessary residues.

CV solutions of different concentrations (1 nM–1 mM) were prepared with each solvent (DI water, 1% human serum, and aquaculture water). The substrates were then immersed in 1 mL of the solution for 30 min and thoroughly dried.

### 2.5. Characterization of AMSP

The morphology of the AMSP was observed using a field-emission scanning electron microscope (FE-SEM; JSM-IT800, JEOL, Tokyo, Japan). SEM images were obtained at 15 kV HV and different magnifications.

Then, the reproducibility and uniformity analyses of AMSP were conducted using 100 μM R6G aqueous solution. Reproducibility analysis involved SERS measurements of the R6G solution in five independently prepared samples. Uniformity analysis involved the measurement of the Raman intensity of R6G at 30 randomly selected locations on the prepared substrate.

## 3. Results and Discussion

### 3.1. Fabrication and Detection Strategy with AMSP

**Figure 1:**
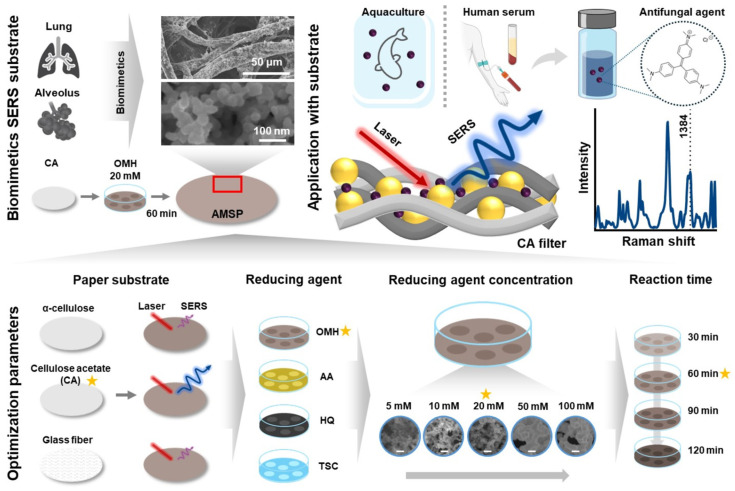
Schematic diagram illustrating the optimization of various parameters for the fabrication of AMSP and their performance evaluation. The optimization parameters included different types of paper, reducing agents, concentrations of reducing agents, and reaction times (Optimization factors are indicated by separate star symbols). Consequently, AMSP, mimicking the structure of lungs and alveoli, was fabricated. These substrates enabled the sensitive detection of CV in various samples. Scale bar in the figure: 100 nm.

The optimization factors and detection strategy for the AMSP used to detect CV are illustrated in Figure 1. The AMSP was designed to mimic the structure of the human alveoli, thereby increasing the surface area available for interaction with air. Gold nanoparticles, known for their rapid and simple synthesis and excellent stability, were chosen as the primary components for this purpose [34]. First, α-cellulose, cellulose acetate, and glass fiber papers were evaluated to determine the appropriate substrate type. Each paper substrate was functionalized with gold nanoparticles and assessed using Raman spectroscopy. Consequently, the SERS substrate based on CA paper showed the highest Raman intensity, and was therefore selected as the optimal substrate. Optimizing the reducing agent in gold nanoparticle synthesis is essential, as each agent’s unique reduction potential influences nanoparticle formation, thereby impacting the substrate’s SERS performance [35]. Gold nanoparticles were functionalized on the selected CA paper substrate using various substances (OMH, AA, HQ, and TSC) as direct reducing agents in the synthesis of gold nanoparticles. The performance of the resulting substrates was evaluated using 100 µM R6G as the Raman reporter. Variations in gold nanoparticle synthesis were expected due to the differing reduction potentials of the reducing agents. The substrate produced using the OMH-reducing agent exhibited the highest Raman intensity, which led to the selection of OMH as the optimal reducing agent. The subsequent optimization focused on the concentration of the OMH-reducing agent and the reaction time. The reducing agent influenced the synthesis of gold nanoparticles, thereby affecting the SERS performance. Similarly, variations in the concentration and reaction time of the same reducing agent affected nanoparticle formation. Various OMH concentrations and reaction times were selected, and performance evaluations were conducted using Raman spectroscopy.

An optimal AMSP for CV detection was fabricated considering the optimized factors. SEM images demonstrated that the resulting substrate successfully mimicked the structure of human alveoli. The increased optical interaction area with the Raman laser, along with the gold nanoparticles arranged at regular intervals in an alveolar-like structure, maximized the SERS effect. This configuration enhanced the Raman signal of the target molecule, leading to improved detection sensitivity.

Using the AMSP detection mechanism, a strategy for the sensitive detection of CV, an antifungal agent in aquatic systems, was developed. Gold nanoparticles adsorbed onto the fiber bundles of the paper formed hotspots. When the target substance was located in these regions, the SERS effect amplified the Raman signal of the target. The performance of the fabricated AMSP was initially evaluated, followed by an assessment of its ability to detect various concentrations of CV, not only under laboratory conditions but also in aquaculture water and serum samples. The specific Raman spectroscopic peaks of the CV appeared at 1170, 1384, and 1583 cm^−1^, with the analysis focusing on the peak at 1384 cm^−1^ [36,37].

### 3.2. Optimization of AMSP

**Figure 2:**
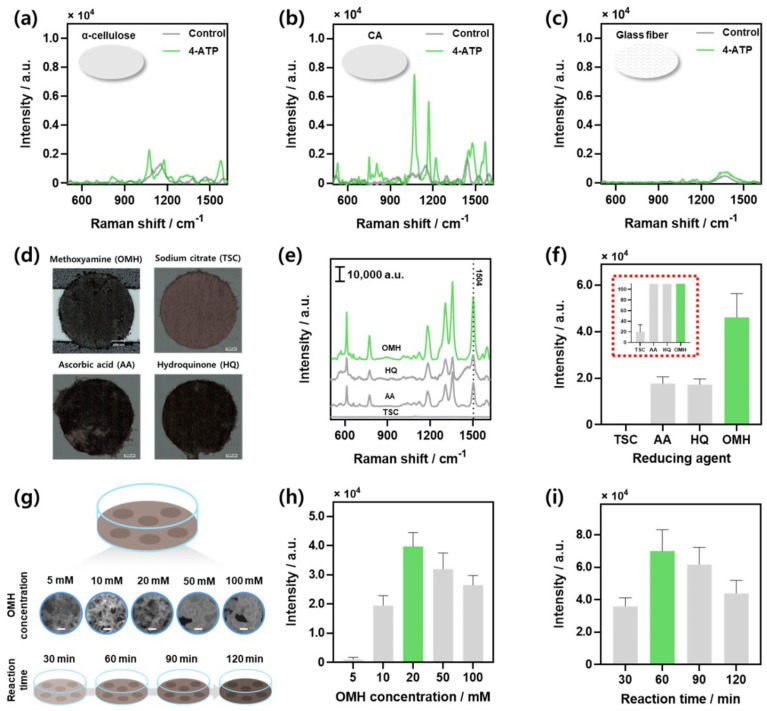
Optimization of paper-based SERS substrates. Raman spectra of 100 μM 4-ATP on SERS substrates made with OMH-reducing agent on different types of paper: (**a**) cellulose, (**b**) CA, and (**c**) glass fiber, respectively. (**d**) Images of SERS substrates prepared on CA paper with different reducing agents (OMH, TSC, AA, HQ). (**e**) Raman spectra of 100 μM R6G reacted on each substrate. (**f**) Comparison of Raman intensities at 1504 cm^−1^ peak of R6G. Red dotted inset image shows data with an adjusted y-axis scale. (**g**) Schematic of optimization process for OMH concentration and reaction time (scale bar: 100 nm). (**h**) Comparison of Raman intensities at 1504 cm^−1^ peak of R6G for AMSPs prepared with varying OMH concentrations. (**i**) Raman intensities at 1504 cm^−9^ peak of R6G for different reaction times using 20 mM OMH-reducing agent.

The AMSP was optimized using two Raman reporters, 4-ATP and R6G (Figure 2). In this experiment, 4-ATP was used to optimize the SERS substrate owing to its high reactivity with amine groups and metal nanoparticles [38]. R6G, a fluorescent dye known to generate strong Raman signals, was used to evaluate its performance on paper-based substrates. Initially, several candidates, including α-cellulose, CA, and glass fiber paper, were assessed to identify the optimal paper substrate for SERS fabrication. Performance was evaluated as outlined in the Materials and Methods section. In the absence of a Raman reporter (control), both the α-cellulose substrate (Figure 2a) and CA substrate (Figure 2b) exhibited similar Raman spectra. However, after the introduction of 4-ATP, the CA substrate exhibited a significantly stronger 4-ATP Raman signal, highlighting its suitability for high-sensitivity applications. Conversely, the glass fiber substrate did not show any 4-ATP Raman signal (Figure 2c). This enhanced performance of the CA substrate can be attributed to its high chemical stability due to acetylation, which not only facilitates the stable physical adsorption of gold nanoparticles but also provides a consistent surface for uniform nanoparticle distribution [39,40]. Unlike CA paper, α-cellulose and glass fibers exhibit relatively high electrostatic properties and irregular surface morphologies, which result in lower and less uniform physical adsorption of nanoparticles. However, glass-fiber substrates, with their limited chemical interaction capabilities, are less effective in promoting the plasmon resonance required for Raman signal enhancement [41,42]. Thus, CA paper, with its acetylated structure, achieves optimal Raman intensity by supporting superior nanoparticle adsorption and plasmonic activity. Consequently, the CA-based substrate exhibited the highest Raman intensity because of its superior nanoparticle adsorption and plasmonic activity.

After confirming that the CA filter paper was the optimal substrate, optimization experiments were conducted to select the most suitable reducing agent for the synthesis of gold nanoparticles. The reducing agents were selected from those commonly used to reduce metal nanoparticles, and the experimental methods are detailed in the Materials and Methods section [32,43,44,45]. Figure 2d illustrates optical microscopy images of the paper-based substrates prepared with each reducing agent.

Performance evaluation was conducted on each substrate using the Raman reporter R6G at a concentration of 100 µM (Figure 2e). The Raman intensity at the characteristic Raman peak of R6G, 1504 cm^−1^, was compared (Figure 2f). The substrate prepared using OMH as the reducing agent exhibited the highest Raman performance. This can be attributed to the relatively low reduction potential of TSC (−0.18 V) under the same conditions, which led to incomplete nanoparticle synthesis and the absence of an observable SERS effect [46]. In contrast, the reduction potentials of AA and HQ (0.5 and 0.8 V, respectively) were comparatively high, resulting in the aggregation of nanoparticles, thereby reducing the effectiveness of the SERS signal [47,48]. These results suggest that the varying reduction strengths of the reducing agents influence the surface potentials of the synthesized nanoparticles, which in turn affect their degree of physical adsorption onto paper substrates [49].

Subsequently, optimization experiments were conducted to determine the optimal concentration and reaction time for the selected OMH-reducing agent. The concentration and reaction time of the reducing agent can be considered critical factors in the synthesis of gold nanoparticles. A schematic of the concentration- and reaction-time-dependent optimization experiments is presented in Figure 2g. Performance evaluations for concentration conditions were consistently carried out using 100 µM R6G, and the results are displayed in Figure 2h. The SEM images of the substrates at varying concentrations are shown in Figure S1. These images include the bare CA paper substrate (control, Figure S1a) and the substrates prepared with OMH concentrations of 5, 10, 20, 50, and 100 mM (Figure S1b–f). No gold nanoparticles were observed in the control sample, whereas physical adsorption of gold nanoparticles onto the paper fibers was noticeable in the substrates treated with the reducing agent. The nanoparticles appeared unevenly distributed on the fibers at lower concentrations; this is likely because of the insufficient concentration of the reducing agent, which impeded proper nanoparticle synthesis [50,51]. Conversely, excessive nanoparticle formation led to aggregation at higher concentrations (50 and 100 mM), resulting in nonuniform adsorption. In contrast, the substrate prepared with 20 mM OMH contained uniformly distributed, well-formed gold nanoparticles. Significant differences in the size and uniformity were observed between the nanoparticles synthesized at 20 and 100 mM (Figure S2). Subsequently, the overall Raman spectra for each condition were examined, as demonstrated in Figure S3. Comparing the Raman intensities at the characteristic peak of R6G at 1504 cm^−1^, the substrate prepared with 20 mM OMH exhibited the highest Raman performance (Figure 2h). This enhanced performance can be attributed to the variation in the gold nanoparticle size, which is influenced by the concentration of the reducing agent, as previously discussed. The observed differences are ascribed to these factors because the SERS performance is maximized through optimized particle size and interparticle gaps.

The reaction times evaluated were 30, 60, 90, and 120 min, representing the duration of the reaction between the reducing agent and HAuCl_4_. The performance evaluation results for each condition are demonstrated in Figure S4a–d with whole Raman spectra. Figure 2i shows the comparison of Raman intensities at the characteristic peak of R6G at 1504 cm^−1^, revealing that the substrate with a 60 min reaction time exhibited the highest Raman intensity. Thus, the optimal reaction time for synthesizing gold nanoparticles with the highest SERS efficiency was 60 min. Shorter reaction times resulted in insufficient nanoparticle formation, limiting the SERS effect. Conversely, extending the reaction time beyond 60 min led to increased particle size and aggregation, thus diminishing the enhancement efficiency [52,53]. Based on these optimization assessments, the final optimized substrate for this study was the substrate that reacted for 60 min using a 20 mM OMH-reducing agent on CA paper.

### 3.3. Performance Evaluation of Optimized AMSP

**Figure 3:**
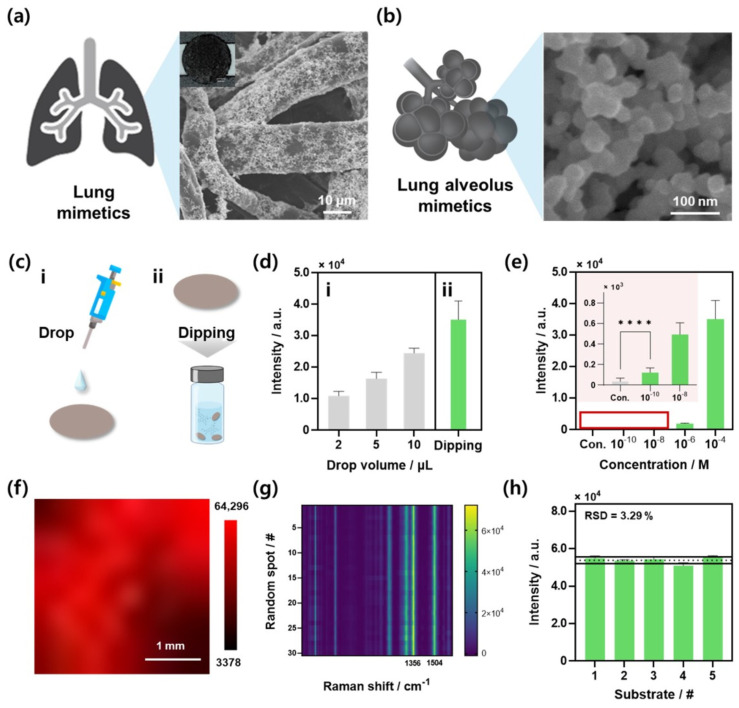
Performance evaluation of the optimized AMSP. (**a**) SEM image of AMSP developed in this study, and (**b**) SEM image of substrate mimicking alveolar structure. (**c**) Schematic of optimization of sample reaction conditions: (i) drop-casting and (ii) dipping. (**d**) Comparison of Raman intensities at 1504 cm^−1^ peak of 100 μM R6G for different reaction conditions: (i) results for drop volumes of 2, 5, and 10 μL, and (ii) results for dipping condition. (**e**) Detection results of R6G at different concen-trations (10^−4^ to 10^−10^ M) under optimized conditions; inset image shows results for low concentrations, with **** *p*-value < 0.0001. (**f**) Raman mapping image of 100 μM R6G indicator on AMSP. (**g**) Heatmap image of Raman spectra at 30 random spots (#: number unit) on same substrate, showing bright colors at specific peaks of 1356 and 1504 cm^−1^. (**h**) Reproducibility assessment across five different AMSPs using measurements of 100 μM R6G indicator; RSD = 3.29%.

Performance evaluations were conducted to assess the suitability of the optimized paper-based substrate as a Raman spectroscopy platform. An actual photograph of the substrate fabricated under the optimized conditions is shown in Figure S5. This substrate utilizes the fiber structure of cellulose acetate paper, which is similar to the structure of the respiratory tract, as shown in Figure 3a. The structure of the trachea maximizes the airflow path, thereby increasing the efficiency of gas exchange within the lungs. Similarly, segmented fiber clusters increase the probability and efficiency of target molecule interactions. Figure 3b shows an SEM image of the gold nanoparticle structure designed to mimic alveoli. By forming and functionalizing nanoparticles on the fiber surface using OMH as a reducing agent, a substrate that mimics the structure of human alveoli is created. Alveoli have evolved to maximize the surface area in contact with capillaries and air, thereby increasing the efficiency of gas exchange. Similarly, gold nanoparticles maximize the surface area to facilitate efficient interactions with target molecules and improve the contact area with the Raman laser, thereby maximizing the SERS signal at the hotspot. Subsequently, the performance was evaluated using the alveoli-mimicking gold nanoparticle-coated cellulose acetate paper substrate (AMSP).

Prior to evaluation, the method for reacting the target substance with AMSP was optimized (Figure 3c). Two methods were compared: dropping the target substance in equal volumes onto the AMSP, allowing it to react before measurement (Figure 3c(i)), and dipping the AMSP into a prepared target substance solution, enabling sufficient reaction time (Figure 3c(ii)). These are the two most common sample preparation methods used for Raman measurements. The drop-casting method improves measurement efficiency by concentrating the target material as the sample dries, leading to localized accumulation [54]. Conversely, the dipping method enhances measurement efficiency by ensuring a uniform interaction between the target material and the entire substrate rather than being confined to specific areas [55]. For comparison, 100 µM R6G was used as the Raman indicator, and the results are shown in Figure 3d. When dropping the same volumes (2, 5, and 10 µL), the Raman intensities were 10,853.82 ± 1401.77, 16,318.33 ± 2022.82, and 2428.35 ± 1596.12, respectively (Figure 3d(i)). Conversely, the Raman intensity after sufficient dipping in the R6G solution and drying was 35,123.81 ± 5881.78 (Figure 3d(ii)). In general, when a sample is applied to a paper-based substrate by drop-casting, an increase in the reaction volume leads to a corresponding increase in the amount of the target, resulting in enhanced Raman intensity. However, this method is limited by an insufficient target loading relative to the surface area of the substrate and the potential for sample leakage beneath the substrate. In contrast, immersing the substrate in the solution allows for more efficient binding of the target material across the substrate, even at the same concentration. Consequently, the dipping method produced superior Raman intensity; thus, all subsequent experiments were performed using this optimized dipping procedure.

The Raman spectroscopic detection performance for R6G was evaluated at various concentrations using the optimized sampling conditions. Detection was conducted across a range from 100 µM to 100 pM, and the comparison of Raman intensities at the characteristic peak of R6G (1504 cm^−1^) is presented in Figure 3e. The inset in Figure 3e shows the scaled data for the control, 100 pM, and 10 nM concentrations. The analysis showed that the control and 100 pM samples passed the *t*-test with a *p*-value < 0.0001, confirming R6G detection at 100 pM. This suggests that, under laboratory conditions using AMSP, the experimental detection limit for CV is 100 pM. Raman spectra for the 10 nM, 100 pM, and control samples are presented in Figure S6, showing the R6G peak at 1504 cm^−1^. Based on these results, the analytical enhancement factor (AEF) of the AMSP was calculated as follows [56]:

$$AEF=\frac{I_{SERS}/C_{SERS}}{I_{OR}/C_{OR}}$$

Here, *I_SERS_* represents the SERS intensity, *I_OR_* denotes the Raman intensity observed on the bare substrate, *C_SERS_* is the concentration of the Raman indicator on the SERS substrate (i.e., the detection limit), and *C_OR_* is the analyte concentration on the bare substrate. The calculated AEF for the AMSP was 5.93 × 10^6^, and the Raman spectra results according to the data used for calculation and the concentration of the indicator R6G used are shown in Figure S7. The substrate demonstrated exceptional detection sensitivity at pM-level concentrations. Notably, its performance surpassed that of other paper-based SERS substrates presented in the literature (Table S1).

The uniformity and reproducibility of the fabricated substrates were evaluated to confirm their suitability for SERS detection. After reacting the R6G indicator with the AMSP, Raman mapping images (10 × 10) were acquired at 100 spots in the defined area (Figure 3f). The higher Raman intensities are represented in red, indicating uniformly high signals across the entire region. Subsequently, the Raman spectra measured at 30 random spots on a single substrate were displayed as a heatmap (Figure 3g). The distinct peaks at 1356 and 1504 cm^−1^ denote the strong SERS response of R6G on the AMSP substrate, with the spectrum color confirmed to be uniform across all measurements. Finally, five different substrates were fabricated and reacted with 100 µM R6G using the same method (Figure 3h). The Raman intensities at 1504 cm^−1^, the specific peak of R6G, were compared across the substrates, showing a relative standard deviation (RSD) value of 3.29%; this demonstrated the excellent uniformity and reproducibility of the substrates.

### 3.4. CV Detection in Various Samples: DI Water, Aquaculture, and Human Serum

**Figure 4:**
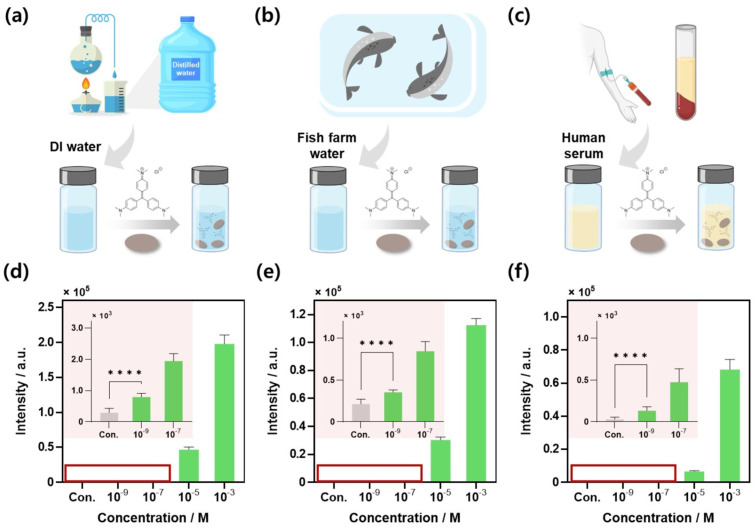
Detection of CV using AMSP. Schematics of sample preparation and reaction conditions for CV detection in (**a**) DI water, (**b**) fish farm water, and (**c**) human serum. Comparison of Raman intensities at 1384 cm^−1^, a specific Raman peak of CV under each condition, (**d**) DI water, (**e**) fish farm water, (**f**) 10% human serum. The inset image of each data is the low-concentration result data of CV with the *y*-axis scale adjusted. (**** *p*-value < 0.0001.)

Environments in which CV detection is critically required include fish farm waters in aquaculture and human biological systems. Therefore, to validate the CV detection capability of the AMSP, experiments were designed to detect CV in fish farm water, human serum samples, and DI water samples in a controlled laboratory setting (Figure 4). Initially, the experiments were conducted using purified DI water samples under laboratory conditions. CV sample solutions were prepared at concentrations ranging from 1 mM to 1 nM using DI water, and each sample was reacted with AMSP by immersion for 30 min (Figure 4a). The Raman intensity results for the specific CV Raman peak (1384 cm^−1^) across different concentrations in DI water samples are shown in Figure 4d. The control and 1 nM concentrations satisfied the *t*-test criteria, with a *p*-value < 0.0001. This result serves as strong evidence that the low control signal highlights the advantages of CA paper and the appropriateness of the experimental approach. The comprehensive Raman spectra for each concentration are presented in Figure S8a-i, while the spectra for lower concentrations (10 μM, 1 nM, and control) are depicted separately in Figure S8a-ii.

Subsequently, water samples were collected from a campus fish farm, filtered, and used to prepare CV solutions at various concentrations. The AMSPs were reacted with each CV sample following the same procedure (Figure 4b). The Raman intensity results for the specific CV peak at 1384 cm^−1^ across various concentrations in the fish farm water samples are displayed in Figure 4e. In these samples, both the control and 1 nM concentration satisfied the *t*-test criteria, with a *p*-value < 0.0001, confirming that CV detection was experimentally successful at a concentration of 1 nM; this was confirmed to be a meaningful result compared with existing CV detection studies in a real environment [57,58]. The comprehensive Raman spectra for each concentration are provided in Figure S8b-i, with the inset showing the fish farm water used for the experiment. The spectra for lower concentrations (10 μM, 1 nM, and control) are displayed separately in Figure S8b-ii.

Finally, human serum samples were prepared by dilution to 10%. CV was then added to these samples to prepare concentrations ranging from 1 mM to 1 nM, and the substrate was allowed to react with the samples using the same method (Figure 4c). A comparison of the Raman intensities of the characteristic peaks for each CV concentration in the serum samples is shown in Figure 4f. The results indicated that CV detection at a concentration of 1 nM was successfully achieved even in serum samples with a *p*-value < 0.0001, satisfying the *t*-test criteria. The overall Raman spectra for each concentration are depicted in Figure S8c-i, and the spectra for low concentrations are shown separately in Figure S8c-ii. Compared to DI water or fish farm water, serum samples contain numerous impurities that can interfere with the reaction of the target substance, resulting in an overall lower Raman signal intensity, including that of the control [59]. Nevertheless, the AMSP demonstrated excellent CV detection performance across various environmental samples, achieving a sensitivity down to a concentration level of 1 nM. Table 1 provides a comparison with various other paper-based SERS substrates used for CV detection, demonstrating that the AMSP exhibits superior performance.

## 4. Conclusions

In this study, we developed a paper-based AMSP for CV detection, drawing inspiration from the ecological structure of bronchial and alveolar tissues in the lungs. The development process involved optimizing several parameters, such as the type of paper, the reducing agent and its concentration, and the reaction time, resulting in a substrate with superior SERS performance. Optimization experiments were conducted for the reaction with the target materials in addition to optimizing the substrate fabrication, demonstrating the high uniformity and reproducibility of AMSP for CV detection. Performance evaluation utilizing the R6G indicator with the proposed AMSP showed that experimental detection was successful at concentrations up to 100 pM. Under controlled laboratory conditions, the substrate could reliably detect CV at a concentration of 1 nM. Furthermore, its performance in real-world settings was evaluated by testing it with samples from fish farm water and human serum, achieving successful CV detection at the 1 nM level in all environments. Consequently, this study successfully developed a simple, optimized, and disposable paper-based SERS platform capable of effectively detecting CV in real-world environmental samples. This platform underscores the potential of the AMSP to provide high sensitivity across various field applications, highlighting its practical utility in varied sensing environments.

## Figures and Tables

**Table 1:** CV detection reference using various paper-based SERS substrates.

Year	Substrate	Detection Limits	References
2019	Au nanocube	217 nM	[60]
2021	Ag nanostructure on paper-based	10 nM	[61]
2022	AuNPs on paper-based	100 nM	[62]
2022	Ag@Ag nanoparticles on paper-based	10 nM	[63]
2023	AgNPs on paper-based	100 nM	[64]
**2024**	**AMSP**	**1 nM**	**This work**

## Data Availability

Data are contained within the article.

## Supplementary Materials

The following supporting information can be downloaded at: https://www.mdpi.com/article/10.3390/bios14120566/s1, Figure S1: Low-magnification SEM images of paper-based substrates fabricated with different concentrations of OMH: (a) control, (b) 5 mM, (c) 10 mM, (d) 20 mM, (e) 50 mM, and (f) 100 mM.; Figure S2: High-magnification SEM images of paper-based substrates fabricated with different concentrations of OMH: (a) 20 mM and (b) 100 mM.; Figure S3: SERS spectra of 100 μM R6G in paper-based substrates fabricated with different concentrations of OMH. Each inset shows an optical image of a paper-based substrate. (a) 5 mM, (b) 10 mM, (c) 20 mM, (d) 50 mM, and (e) 100 mM.; Figure S4: SERS spectra of 100 μM R6G in paper-based substrates fabricated with different reaction times. Each inset shows an optical image of a paper-based substrate. (a) 30 min, (b) 60 min, (c) 90 min, and (d) 120 min.; Figure S5: Actual image of the AMSP.; Figure S6: Raman spectra data with y-axis scale adjusted for control, 100 pM, and 10 nM concentrations of the R6G indicator.; Figure S7: Raman spectra data with y-axis scale adjusted for control, 100 pM, and 10 nM concentrations of the R6G indicator.; Table S1: Comparison of enhancement factors (EF) of various fabricated paper-based SERS substrates.; Figure S8: Raman spectra of CV at various concentrations. (a-i) Raman spectra of CV in DI water at concentrations ranging from 10^−3^ to 10^−9^ M. (a-ii) Detailed Raman spectra for low concentrations of CV (10^−7^, 10^−9^ M) and control in DI water. (b-i) Raman spectra of CV in fish farm water at concentrations ranging from 10^−3^ to 10^−9^ M, inset image shows water from aquaculture farm used in the experiment. (b-ii) Detailed Raman spectra for low concentrations of CV (10^−7^, 10^−9^ M) and control in fish farm water. (c-i) Raman spectra of CV in 1% human serum at concentrations ranging from 10^−3^ to 10^−9^ M. (c-ii) Detailed Raman spectra for low concentrations of CV (10^−7^, 10^−9^ M) and control in 10% human serum. Refs. [65,66,67] are cited in Supplementary Materials.
