# Supplementary material for: Optimization of Paper-Based Alveolar-Mimicking SERS Sensor for High-Sensitivity Detection of Antifungal Agent
Source: Biosensors (Basel). 2024 Nov 22;14(12):566. doi: 10.3390/bios14120566 (PMC11674341; doi:10.3390/bios14120566)
Supplement: Supplementary file 1 [file biosensors-14-00566-s001.zip › biosensors-3267037-supplementary.pdf]

Optimization of Paper-based Alveolar-Mimicking SERS Sensor for High-Sensitivity Detection of Antifungal Agent

Hyunjun Park[a,1], Kyunghwan Chai[b,1], Eugene Park[a,1], Woochang Kim[a], Gayoung Kim[b], Joohyung Park [a,*], Wonseok Lee [c,*] and Jinsung Park[a,d,*]

[a] *Department of Biomechatronic Engineering, College of Biotechnology and Bioengineering, Sungkyunkwan University, Suwon 16419, Korea*

[b] *Department of Biopharmaceutical Convergence, Sungkyunkwan University, Suwon 16419, Korea*

[c] *Department of Electrical Engineering, Korea National University of Transportation, Chungju 27469, Republic of Korea*

[d] *Department of MetaBioHealth, Sungkyunkwan University, Suwon, 16419, Republic of Korea*

*** Corresponding author**

E-mail address: parkjoodori@gmail.com (J. Park), wslee@ut.ac.kr (W. Lee), and nanojspark@skku.edu (J. Park)

Author contributions

[1]These authors contributed equally to this work.

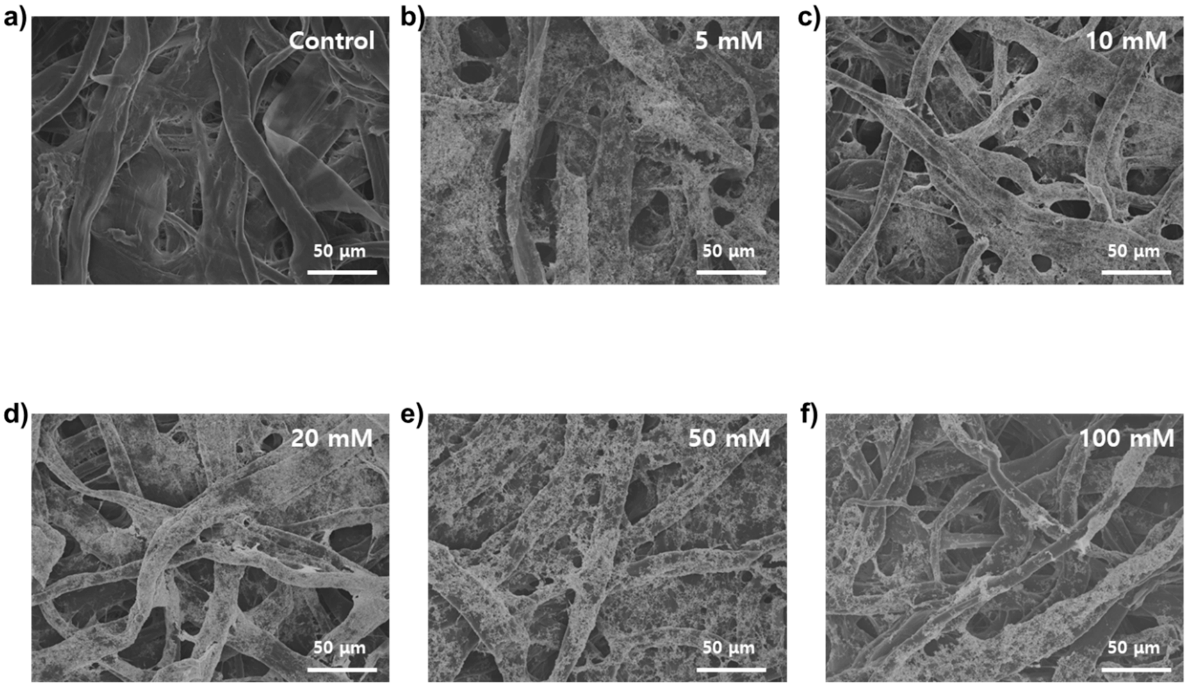

Figure S1. Low-magnification SEM images of paper-based substrates fabricated with different concentrations of OMH: (a) control, (b) 5 mM, (c) 10 mM, (d) 20 mM, (e) 50 mM, and (f) 100 mM.

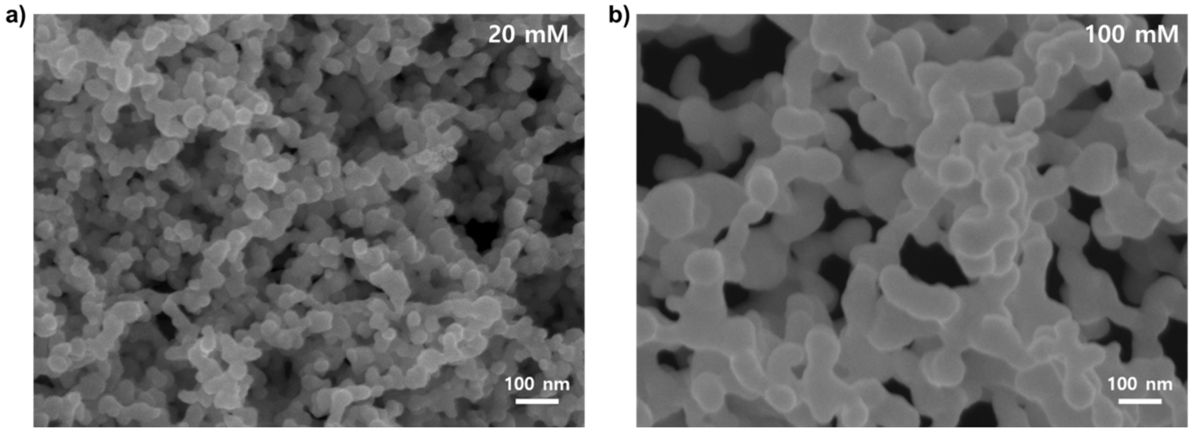

Figure S2. High-magnification SEM images of paper-based substrates fabricated with different concentrations of OMH: (a) 20 mM and (b) 100 mM.

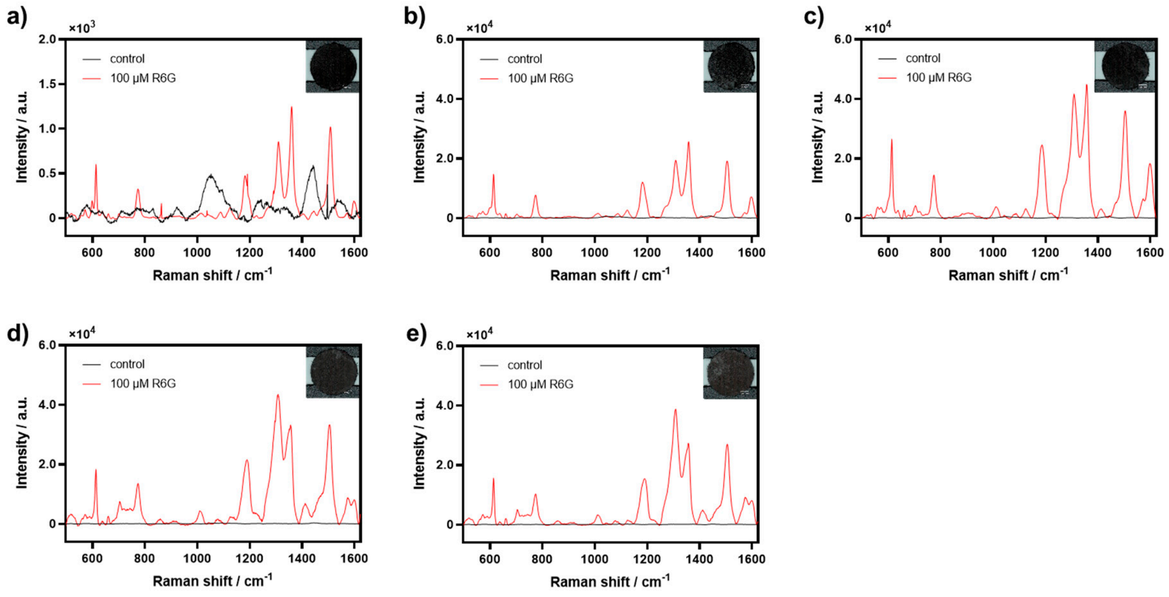

Figure S3. SERS spectra of 100 μM R6G in paper-based substrates fabricated with different concentrations of OMH. Each inset shows an optical image of a paper-based substrate. (a) 5 mM, (b) 10 mM, (c) 20 mM, (d) 50 mM, and (e) 100 mM.

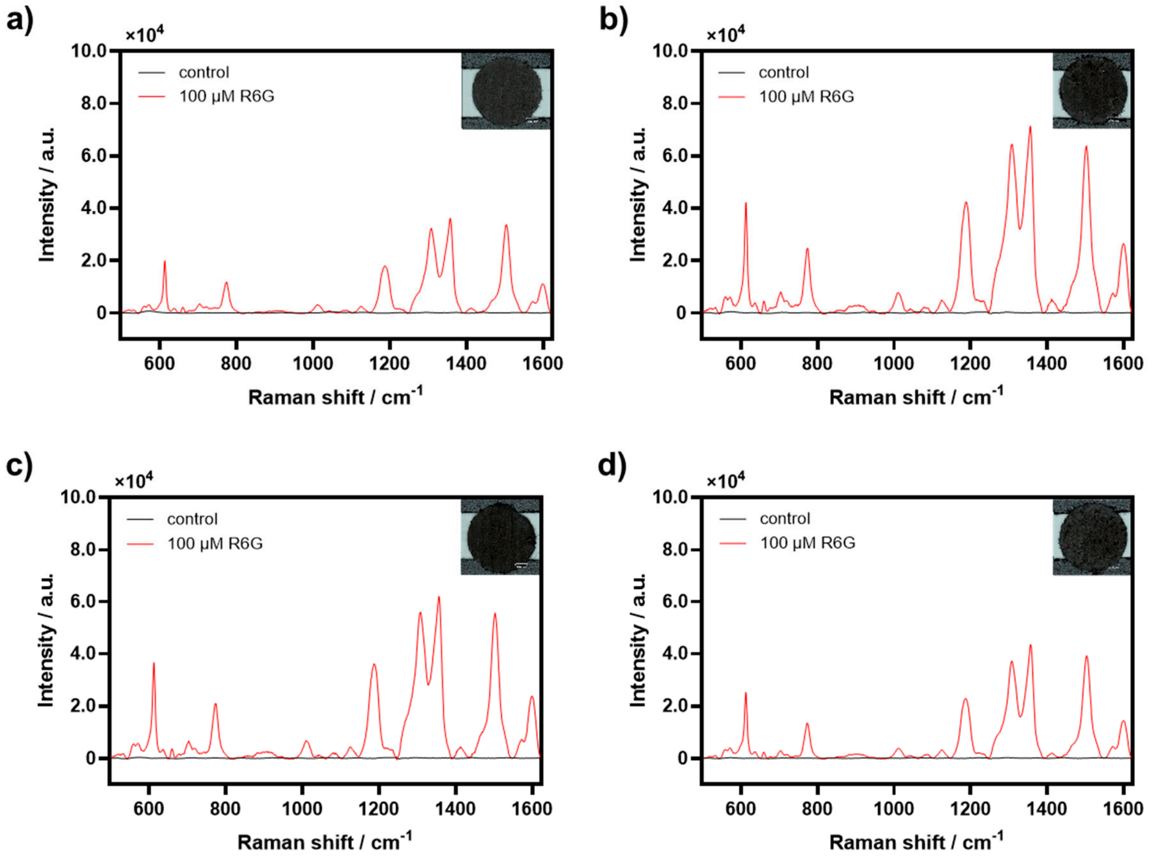

Figure S4. SERS spectra of 100 μM R6G in paper-based substrates fabricated with different reaction times. Each inset shows an optical image of a paper-based substrate. (a) 30 min, (b) 60 min, (c) 90 min, and (d) 120 min.

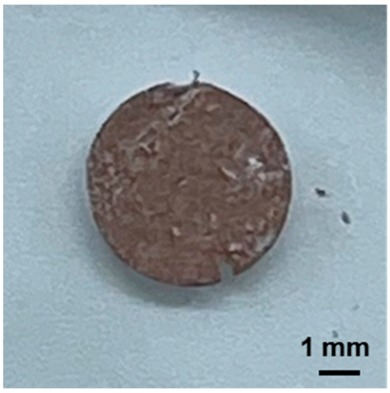

Figure S5. Actual image of the AMSP.

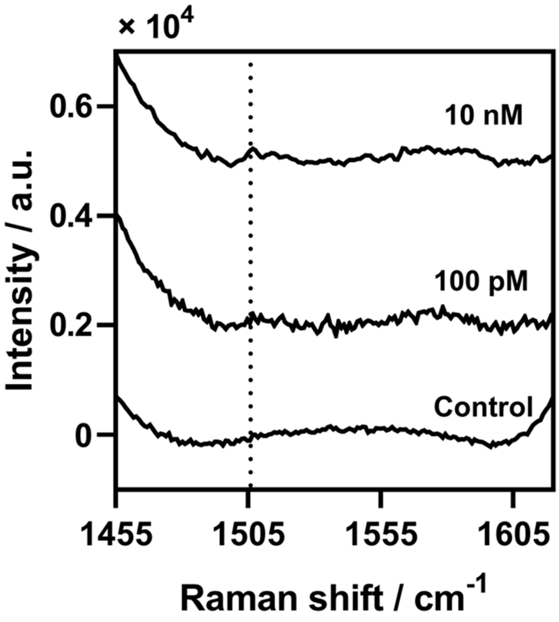

Figure S6. Raman spectra data with y-axis scale adjusted for control, 100 pM, and 10 nM concentrations of the R6G indicator.

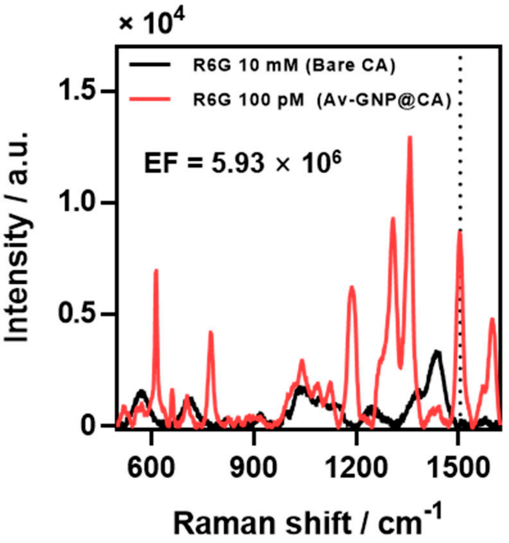

Figure S7. Raman spectra data with y-axis scale adjusted for control, 100 pM, and 10 nM concentrations of the R6G indicator.

Year	Substrate	Enhancement factor (EF)	References
2021	Silver nanowire	**2.63×10^6**	[65]
2021	Ag nanostructure	**1.2×10^5**	[61]
2022	Ag@Au nanoparticles	**9.28×10^4**	[63]
2023	Paper-tip	**1.15×10^5**	[66]
2023	AgNP	**3.058×10^5**	[67]
2024	AMSP	**5.93×10^6**	This work

Table S1. Comparison of enhancement factors (EF) of various fabricated paper-based SERS substrates.

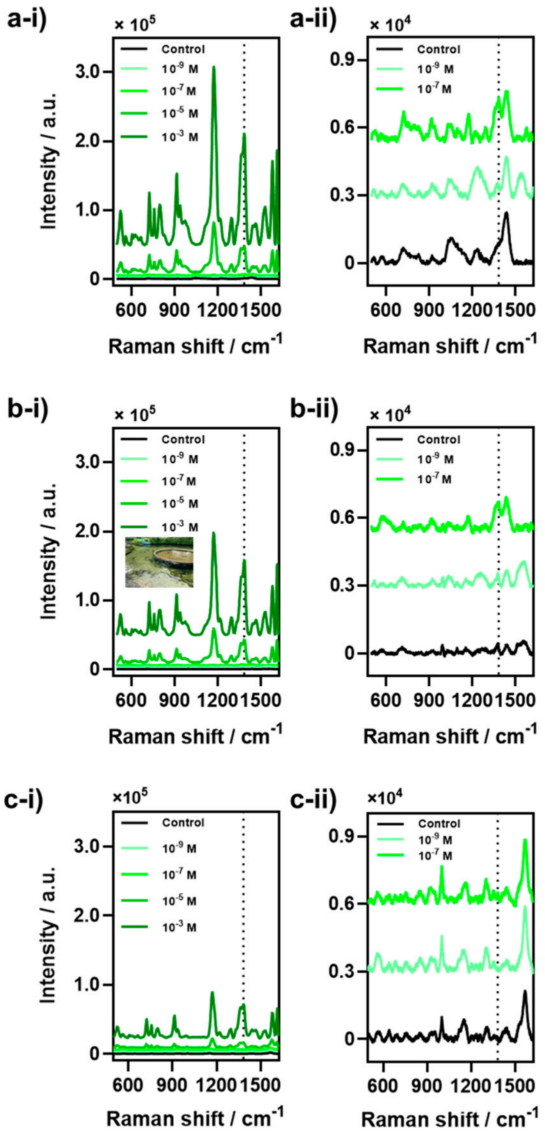

Figure S8. Raman spectra of CV at various concentrations. (a-i) Raman spectra of CV in DI water at concentrations ranging from 10^{-3} to 10^{-9} M. (a-ii) Detailed Raman spectra for low concentrations of CV (10^{-7}, 10^{-9} M) and control in DI water. (b-i) Raman spectra of CV in fish farm water at concentrations ranging from 10^{-3} to 10^{-9} M, inset image shows water from aquaculture farm used in the experiment. (b-ii) Detailed Raman spectra for low concentrations of CV (10^{-7}, 10^{-9} M) and control in fish farm water. (c-i) Raman spectra of CV in 1% human serum at concentrations ranging from 10^{-3} to 10^{-9} M. (c-ii) Detailed Raman spectra for low concentrations of CV (10^{-7}, 10^{-9} M) and control in 10% human serum.
